# Comparison between combined suprainguinal fascia iliaca compartment block with intra-articular bupivacaine and suprainguinal fascia iliaca compartment block in total hip arthroplasty: randomized double blinded controlled trial

**DOI:** 10.1186/s12871-025-03345-y

**Published:** 2025-09-29

**Authors:** Lalisa Saeaeh, Theerawat Chalacheewa, Chatchawat Lerdwimonsak, Sudarat Phurikamonarunothai

**Affiliations:** Department of Anesthesiology, Faculty of Medicine Ramathibodi Hospital, Mahidol University, 270 Rama VI Rd, Ratchathewi, Bangkok, 10400 Thailand

**Keywords:** Fascia iliaca compartment block, Intra-articular bupivacaine, Analgesic efficacy, Postoperative pain management, Morphine consumption, Total hip arthroplasty, Pain scores and patient satisfaction

## Abstract

**Background:**

Hip innervation is complex, with contributions from the lumbar plexus and sacral plexus. In any surgical approach, the nerves are affected in both the lumbar and sacral plexus. Fascia iliaca compartment block (FICB) could be effective analgesia in total hip arthroplasty (THA), but some patients require morphine for breakthrough pain. This may be due to FICB only blocking the anterolateral aspect of the hip joint, failing to block the posterior aspect. However, blocking the sacral plexus may lead to adverse effects, such as delayed ambulation and the risk of foot drop. Intra-articular (IA) injection of local analgesics after joint capsule closure is effective in relieving postoperative pain. We hypothesized that combined FICB with IA bupivacaine would enhance analgesic efficacy compared with FICB alone.

**Methods:**

Twenty-six patients undergoing primary total hip arthroplasty under spinal anesthesia were randomly allocated to receive either a combined suprainguinal fascia iliaca compartment block (S-FICB) with IA (*n* = 13), using 40 ml of 0.25% bupivacaine in the S-FICB and 0.5% bupivacaine injected into the intraarticular hip joint after surgical wound closure, or S-FICB alone (*n* = 13), using 40 ml of 0.25% bupivacaine. Following the block administration, a blinded observer recorded cumulative breakthrough morphine consumption, pain scores at 0, 6, 12, 24, and 48 h, opioid-related side effects, as well as the length of hospital stay and patient satisfaction.

**Results:**

Compared with S-FICB with IA and S-FICB alone, there was no statistic significant in morphine consumption in 0 h (*P* = 0.960), 6 h (*P* = 0.169), 12 h (*P* = 0.125), 24 h (*P* = 0.186), and 48 h (*P* = 0.311) in this study. No statistic significant were found in secondary outcome in term of static and dynamic NRS scores, incidence of nausea and vomiting, block related LAST and patient satisfaction.

**Conclusion:**

The addition of 20 ml of 0.5% intra-articular bupivacaine to a suprainguinal fascia iliaca compartment block did not provide additional postoperative analgesic benefits for patients undergoing total hip arthroplasty.

## Introduction

Hip innervation is complex with contributions from lumbar plexus that innervate anterolateral area of hip joint and sacral plexus that innervate posterior aspect of hip joint [1]. Fascia iliaca compartment block (FICB) provides an indirect proximal approach to the lumbar plexus and provides sensory blockade to several of the nerves that supply anterolateral aspect of hip joint. However, FICB is volume dependent block so some FICB patients require morphine for breakthrough pain [2–4]. This may be due to FICB only blocks the anterolateral aspect of the hip joint, failing to block the posterior aspect, resulting in persistent pain for the patients which is a similar finding in our institute. However, blocking the sacral plexus may lead to adverse effects, such as delayed ambulation and the risk of foot drop in patients.

Intra-articular (IA) injection of local analgesics is one pain control techniques because local anesthetic block pain conduction at its origin. Studies examining administration of intra-articular bupivacaine in arthroscopic knee surgery and after joint arthroplasty were effective for pain pain relief [5, 6].

The purpose of this study was to compare a combined suprainguinal fascia iliaca compartment block (S-FICB) with IA and the S-FICB alone in total hip arthroplasty. We hypothesized that S-FICB with IA would reduce opioid requirement than S-FICB alone.

## Methods

### Study design

This study received approval as a randomized, double-blind, placebo-controlled trial from the Ramathibodi Hospital, Mahidol University Institutional Review Board (COA. MURA2021/726, Aug 18, 2021) before initiating patient enrollment. All participants provided informed written consent before inclusion in the study.

### Participants

After obtaining ethical approval, all patients scheduled for total hip arthroplasty (THA) underwent eligibility assessment. Twenty-eight patients were approached, and twenty-six provided consent to participate in the study. Two participants were excluded—one due to chronic opioid use and another due to a local skin infection at the lower back. Inclusion criteria encompassed patients undergoing elective primary THA under spinal anesthesia with American Society of Anesthesiologists (ASA) physical status I to IV. Exclusion criteria comprised refusal, contraindication to local anesthesia, chronic pain or opioid use exceeding 2 weeks within the past month, local skin infection at the injection site, Body Mass Index (BMI) > 40 kg/m2, renal impairment (Creatinine Clearance < 30 mg/ml), coagulopathy (platelets < 100,000/mL or INR > 1.4), inability to assess pain scores, inability to operate intravenous patient-controlled analgesia (IV-PCA), and contraindications to spinal anesthesia.

### Sample size calculation

In the pilot study, the mean postoperative 24-hour morphine consumption for the combined S-FICB with IA Bupivacaine group was 1 mg (SD 4 mg), while the S-FICB group was 6 mg (SD 4 mg). Considering a type-1 error of 0.05 and a power of 80%, the initial sample size calculation indicated a minimum of 11 patients per group. Accounting for a 20% dropout rate, the adjusted sample size increased to 13 patients per group. Consequently, the total sample size for this study is set at 26 patients.

### Randomization and blinding

The participants were randomly assigned to receive S-FICB with IA bupivacaine or S-FICB alone through computer-generated random numbers with a 1:1 allocation ratio. Allocation concealment was maintained by an uninvolved assistant using sequentially numbered, sealed, opaque envelopes, opened just prior to the operation. To ensure blinding, the bupivacaine and saline solutions, prepared by the Nurse Surgical Assistant, were visually indistinguishable, rendering the surgeon and anesthesiologist team unaware of the administered solution. Data collection was conducted in a double-blinded manner, ensuring neither patients nor healthcare personnel were cognizant of the medication assignment. The confidentiality of the randomization code was maintained until all data were available for analysis.

### Anesthesia procedure

Following standard monitoring, patients were cannulated with an 18G intravenous catheter. Pre-medication included intravenous midazolam (2–5 mg) and/or fentanyl (25–50 mcg) administered before S-FICB. All participants assumed a supine position for the block technique. S-FICB initiation involved placing the Ultrasound (US) transducer longitudinally at the level of the anterior superior iliac spine (ASIS), following the technique described by Desmet et al. [7]. Sliding the US transducer in a medial and caudal direction allowed identification of the iliacus muscle (IM) and fascia iliaca (FI). To achieve the ‘bow-tie sign,’ the transducer was slightly rotated, positioning the cranial end toward the umbilicus and the caudal end at the ASIS in the parasagittal plane, facilitating the identification of the deep circumflex iliac artery (DCIA). The needle, introduced in-plane from caudal to cranial under US guidance, reached the FI’s level at the DCIA. A successful injection, verified by hydrodissection between the FI and IM and cranial spread of local anesthetic (LA) under the FI, involved the administration of 40 mL of 0.25% bupivacaine. Repositioning of the needle was permitted during injection to ensure adequate spread.

Immediately after S-FICB placement, all patients underwent spinal anesthesia in the lateral position. The intervertebral spaces of L3-4 and L4-5 were identified, and a midline or paramedian bupivacaine isobaric solution was administered using a 25–27 gauges Quincke needle.

### Surgical procedure

THA was conducted using a modified anterolateral approach. A skin incision was created in a slightly posterior curved shape, extending from 6 cm above the greater trochanter along the femoral course to 6 centimeters distal to the greater trochanter. After the final prosthetic component was inserted, a surgical suction drainage tube was introduced into the intra-articular area. Following closure of the anterior hip capsule, subcutaneous layer, and skin, an intra-articular injection of 20 ml of 0.5% bupivacaine (100 mg) in the study group or 20 ml of 0.9% normal saline in the control group was administered through the surgical drain. The surgical drain was subsequently discontinued after the injection.

### Analgesia protocol

All patients received uniform postoperative pain management. During the initial 48 h, postoperative pain management consisted of Intravenous Patient-Controlled Analgesia (IV-PCA) morphine (PCA dose of 1 mg, with a lockout interval of 5 min and a 4-hour limit of 40 mg), acetaminophen (500 mg) administered orally once after meals four times a day, and either etoricoxib (90 mg) or celecoxib (400 mg) administered orally once daily.

### Outcome assessment

The primary outcome was postoperative 24-hour morphine consumption. The secondary outcomes measured were postoperative pain score at rest and on movement (NRS 0–10) at post anesthesia care unit (PACU), 6, 12, 24, 48 h, time to first morphine requirement was recorded as time zero at PACU, incidence of Nausea and vomiting was defined as mild: no treatment needed, moderate: 1–2 dose of antiemetics, severe: require > 2 dose of antiemetic, block related complications including local anesthetic systemic toxicity (LAST), patient satisfaction on overall postoperative pain management as score 0–10, length of hospital stay as days.

### Other measurement data

Demographic data, including gender, age, weight, height, BMI, underlying disease, diagnosis, ASA physical status I to IV, operation time, and blood loss were systematically recorded. Patients were visited in the ward until 48 h postoperatively to inquire about the presence of persistent sensory (numbness) or motor (weakness) deficits.

### Statistical analysis

Statistical analyses were conducted utilizing Stata, version 20, with numerical data presented as mean and standard deviation (SD) or median and range, as appropriate. Qualitative data were expressed in terms of frequency and percentage. The T-test was employed for normally distributed continuous data, while the Mann-Whitney test was utilized for non-normally distributed variables. Categorical data were compared using either Pearson’s Chi-square test or Fisher’s exact test. A significance threshold of p-value less than 0.05 was considered statistically significant.

## Results

**Fig. 1:**
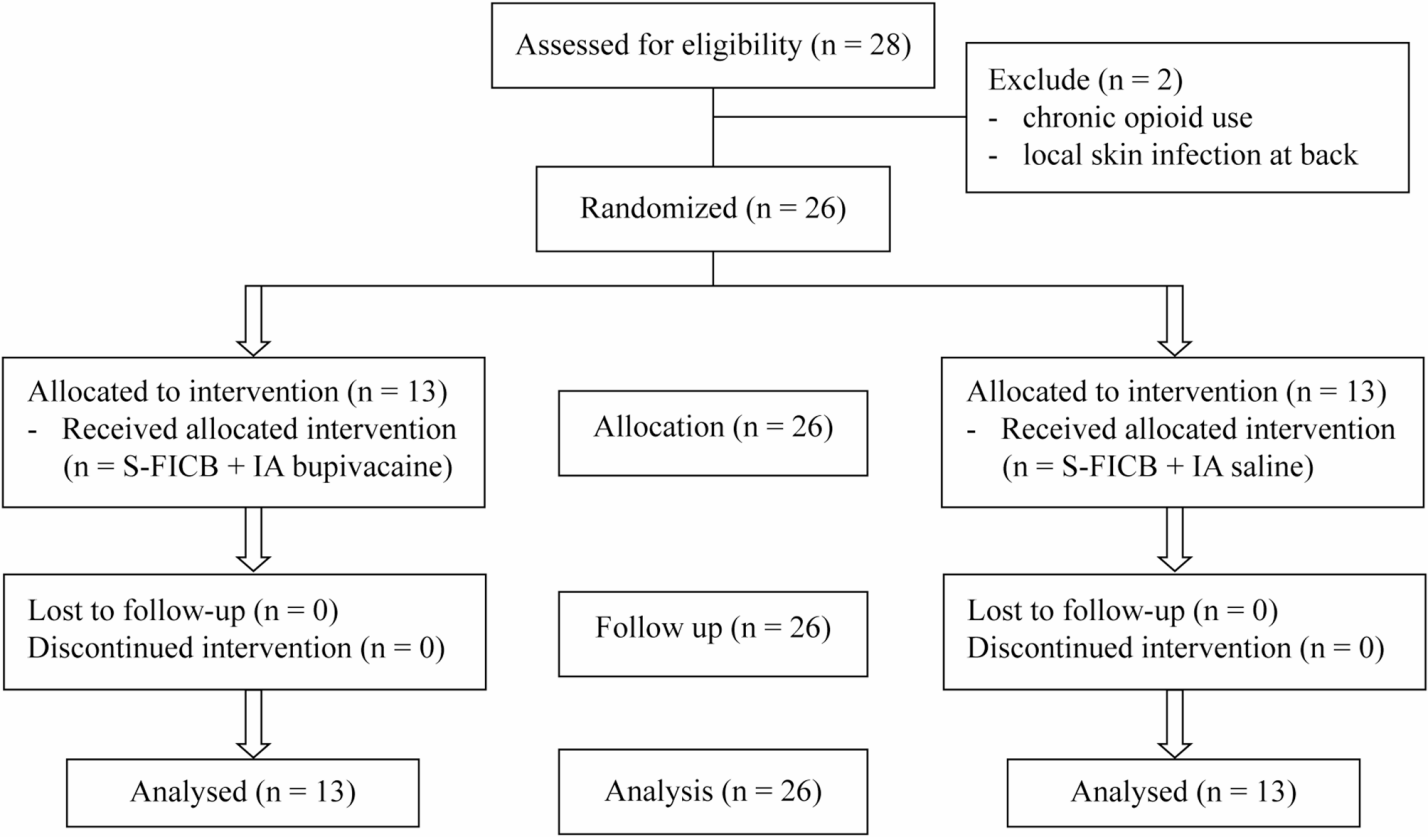
Consort diagram of the study

A total of twenty-eight patients were enrolled in the study, as shown in Fig. 1. However, two participants were subsequently excluded, one due to chronic opioid use and another due to a local skin infection at the lower back. The remaining twenty-six patients constituted the randomized controlled trials cohort. The demographic characteristics of these patients are shown in Table 1, revealing no statistically significant differences between the two groups.

**Table 1:** Patient characteristics

Patient Characteristics	S-FICB with IA (*n* = 13)	S-FICB (*n* = 13)	*P*- value
Age (years)	60.62 ± 14.04	64 ± 11.65	0.510
Sex			
Female	3 (23.08%)	4 (30.77%)	> 0.999
Male	10 (76.92%)	9 (69.23%)	
Weight (kg)	64.77 ± 14	65.85 ± 15.24	0.853
Height (cm)	157.08 ± 5.62	155.77 ± 7.34	0.615
Body Mass Index (kg/m^2^)	26.15 ± 5.35	26.77 ± 4.94	0.763
Underlying disease			
HT	6 (46.15%)	7 (53.85%)	0.695
DM	3 (23.08%)	2 (15.38%)	> 0.999
DLP	9 (69.23%)	7 (53.85%)	0.420
CKD	1 (7.69%)	3 (23.08%)	0.593
Other	7 (53.85%)	8 (61.54%)	0.691
Diagnosis			
OA Hip	9 (69.23%)	11 (84.62%)	0.645
AVN	4 (30.77%)	2 (15.39%)	
ASA			
1	1 (7.69%)	0 (0%)	0.687
2	8 (61.54%)	7 (53.85%)	
3	4 (30.77%)	6 (46.15%)	
Operation time (minutes)	195 (180–240)	180 (165–180)	0.113
Surgical time (minutes)	143 (120–150)	135 (120–150)	0.840
Blood loss (mL)	400 (300–750)	400 (300–500)	0.448
Intraoperative complication	0 (0%)	0 (0%)	
PACU time (minutes)	60	60.77 ± 2.77	0.762
Duration of sensory block (hours)	34.08 ± 4.44	38 ± 9.06	0.174

Data are expressed as mean ± standard deviation or Median (Min – Max) or n (%)

*Abbreviations*: *OA* Osteoarthritis, *HT* Hypertension, *DM* Diabetes mellitus, *DLP* Dyslipidemia, *CKD* Chronic Kidney Disease, *AVN* Avascular necrosis, *ASA* American Society of Anesthesiologists, *PACU* Post Anesthesia Care Unit

The primary outcome, postoperative 6, 12, 24 and 48-hour morphine consumption, is shown in Table 2. Analysis revealed no significant differences between the two groups in cumulative morphine consumption at 0, 6, 12, 24, and 48 h after surgery

**Table 2:** Postoperative cumulatively milligrams morphine consumption

Morphine Consumption (mg)	S-FICB with IA (*n* = 13)	S-FICB (*n* = 13)	*P*- value
PACU 6 h 12 h 24 h 48 h	0 (0–0) 3 (1–5) 5 (4–11) 10 (6–14) 11 (8–18)	0 (0–0) 1 (1–3) 3 (2–8) 6 (4–8) 8 (7–10)	0.960 0.169 0.125 0.186 0.311

Data are expressed as Median (Min – Max)

Secondary outcomes, No statistic differences in postoperative NRS scores at rest and on movement during PACU, as well as at 6, 12, 24, and 48 h after surgery between groups. Additional time to first morphine requirement, the incidence of nausea and vomiting, block-related local anesthetic systemic toxicity (LAST), patient satisfaction with overall postoperative pain management, and length of hospital stay were not statistic significant differences between the groups shown in Table 3

**Table 3:** Secondary outcome

Secondary outcomes	S-FICB with IA (*n* = 13)	S-FICB (*n* = 13)	*p*-value
NRS rest			
PACU	0 (0–0)	0 (0–0)	0 (0–0)
6 h	2 (1–5)	2 (1–3)	
12 h	3 (1–4)	1 (1–2)	
24 h	1 (0–2)	2 (1–3)	
48 h	2 (0 - 2)	1 (0–2)	
NRS movement			
PACU	0 (0–1)	0 (0–1)	0.584
6 h	4 (2–7)	4 (2–5)	
12 h	3 (2–4)	4 (3–4)	
24 h	3 (2–4)	4 (3–4)	
48 h	3 (2–3)	3 (2–4)	
Time to first Morphine (hours)	1 (0–2)	4 (1–5)	0.091
N/V			
Yes	1 (7.69%)	0 (0%)	> 0.999
No	12 (92.31%)	13 (100%)	
LAST			
Yes	0 (0%)	0 (0%)	> 0.999
No	13 (100%)	13 (100%)	
Patient satisfaction			
10	12 (92.31%)	11 (84.62%)	> 0.999
8	1 (7.69%)	2 (15.38%)	
Hospital stay (days)	4 (3–5)	4 (4–5)	0.724

Data are expressed as Median (Min – Max) or n (%)

*Abbreviations*: *N/V* Nausea and vomiting, *LAST* Local anesthetic systemic toxicity

## Discussion

This study found that the addition of intra-articular bupivacaine to a suprainguinal fascia iliaca compartment block did not result in a statistically significant reduction in postoperative morphine consumption or pain scores for patients undergoing total hip arthroplasty.

Although there have been numerous reports of intra-articular bupivacaine showing effectiveness for pain relief after joint arthroplasty, the pain relief effect from local injection of bupivacaine is likely related to the degree of proximity of the injection site to the soft tissue around the hip joint. We believe that this factor may have contributed to the non-significant outcomes in this study. Chen et al. [8] used a 0.5% bupivacaine 60 ml intra-articular injection, which reduced postoperative pain and decreased the amount of meperidine required during the first 12 h after THA surgery. Another study by Chen et al. [9], which administered an intra-articular infusion of 0.5% bupivacaine at a rate of 2 ml/h for 48 h and found no pain relief. Lunn et al. [10] reported that intra-operative infiltration of 150 ml of 0.2% ropivacaine during THA failed to provide pain relief. Aguirre et al. [11] administered a 0.3% ropivacaine 20 ml bolus before wound closure, followed by an infusion of 8 ml/h of 0.3% ropivacaine, which provided pain relief. However, a preliminary study by Aguirre et al. using 0.2% ropivacaine failed to provide an analgesic effect. These previous studies have also provided conflicting results regarding intra-articular local anesthetic dose, whether increasing concentration, adding a bolus, or using continuous infusion after a bolus. However, in our study, we used only 20 ml of 0.5% bupivacaine for intra-articular injection due to concerns about local anesthetic systemic toxicity (LAST). This limited volume may have been insufficient to provide optimal analgesia. The total dose of bupivacaine administered (100 mg for intra-articular injection and 100 mg for S-FICB) in patients with a mean body weight of 65 kg slightly exceeded the 3 mg/kg threshold. Nevertheless, all patients were closely monitored for signs of LAST, and no adverse events were observed.

This study employed multimodal analgesia, including FICB with perioperative paracetamol and COX-2 inhibitors, following the PROSPECT guideline for total hip arthroplasty [12]. As a result, this approach provided effective analgesia for THA, as evidenced by our findings of no statistically significant differences in morphine requirements or NRS scores between the two groups.

Our study was a randomized, double-blind trial. The randomization process was validated by statistical analysis and resulted in matched demographic data for patients undergoing THA with a standardized anesthesiologist performing the FICB. However, the study had limitations, including the involvement of three different surgeons performing the THA, which may have resulted in variations in surgical techniques and tissue trauma. Additionally, the number of research participants may not have been sufficient to definitively answer the research questions.

## Conclusion

In patients undergoing total hip arthroplasty, combining a suprainguinal fascia iliaca compartment block with a 20 ml intra-articular injection of 0.5% bupivacaine does not appear to offer superior postoperative pain relief compared to the suprainguinal fascia iliaca compartment block alone.

## Data Availability

No datasets were generated or analysed during the current study.
